# Advanced glycation end products induce inflammaging in periodontal ligament fibroblasts through RAGE/AKT/mTOR/glycolysis pathway

**DOI:** 10.2340/aos.v84.44581

**Published:** 2025-08-21

**Authors:** Lin Xiong, Jiayu Shu, Hongli Gao, Yufeng Qin, Yuehan Zhang, Xuelian Chang, Qiang Dong, Helin Chen

**Affiliations:** a College of Stomatology of Guizhou Medical University, Guiyang, China; b Department of Prosthodontics, Stomatological Hospital of Guizhou Medical University, Guiyang, China

**Keywords:** periodontitis, advanced glycation end products, glycolysis, inflammaging, Akt/mTOR pathway

## Abstract

**Background:**

Inflammaging plays a pivotal role in the pathogenesis of multiple age-related diseases, including periodontitis. Advanced glycation end products (AGEs) are known to induce inflammaging and exacerbate periodontitis. However, the mechanisms by which AGEs promote inflammaging remain unclear. This study aimed to investigate the mechanisms underlying AGE-induced inflammaging.

**Methods and results:**

Human periodontal ligament fibroblasts (hPDLFs) were extracted and stimulated with lipopolysaccharide (LPS), with prior treatment using AGEs. The expression of pro-inflammatory cytokines was measured to explore the role of AGEs in LPS-induced inflammation. Subsequently, hPDLFs were treated with AGEs and pre-incubated with 2-deoxyglucose (2-DG, a glycolysis inhibitor), Ly294002 (an AKT/mTOR pathway inhibitor), and FPS-ZM1 (a receptor for advanced glycation end product [RAGE] antagonist) to assess the levels of inflammaging markers, glycolysis, AKT/mTOR pathway activation, and RAGE expression, along with the potential relationships among these factors. Our findings demonstrated that AGEs significantly increased the expression of pro-inflammatory cytokines in response to LPS stimulation. Additionally, AGEs alone elevated the levels of inflammaging factors, including cell senescence, senescence-associated secretory phenotype factors, SA-β-Gal expression, glycolysis markers, and AKT/mTOR pathway activation. Furthermore, inhibiting glycolysis reduced AGE-induced inflammaging, while blocking the AKT/mTOR pathway, suppressed both AGE-induced inflammaging and glycolysis. Antagonizing RAGE effectively blocked AGE-induced inflammaging, glycolysis, and AKT/mTOR pathway activation.

**Conclusions:**

Our study indicated that AGE-induced inflammaging through binding to RAGE to activate the AKT/mTOR pathway and eventually enhancing glycolysis level, which may contribute to the increased inflammatory response triggered by LPS. These findings suggest that inflammaging is a critical mechanism through which AGEs exacerbate periodontitis.

## Introduction

Periodontitis is a chronic infectious disease affecting periodontal tissues [1], with a global prevalence rate of approximately 45–50%, and this figure continues to rise [2]. Currently, periodontitis ranks as one of the most prevalent diseases worldwide and is a leading cause of tooth loss in older adults [3]. Numerous systemic diseases, such as diabetes, can aggravate periodontitis. There is emerging evidence to support the existence of a two-way relationship between diabetes and periodontitis, with diabetes increasing the risk for periodontitis, and periodontal inflammation negatively affecting glycaemic control [4]. Diabetes mellitus exacerbates periodontitis, with advanced glycation end products (AGEs) identified as a significant contributing factor [5, 6]. AGEs, a heterogeneous group of compounds, interact with the receptor for advanced glycation end products (RAGE) to modulate cellular metabolism [7, 8]. However, the precise mechanisms by which AGEs exacerbate periodontitis remain incompletely understood.

Periodontitis is an age-related disease, in which cellular senescence plays a significant role in its progression [9]. Inflammaging, a chronic state of low-grade inflammation, is a distinct form of senescence that affects the aging process and contributes to various age-related diseases [10]. Cellular senescence and the senescence-associated secretory phenotype (SASP) are key features of inflammaging [11]. Under certain stimuli, the low-grade inflammatory state can escalate into a more severe inflammatory response, resulting in increased tissue damage. Consequently, inflammaging is implicated in the pathogenesis of numerous inflammatory diseases, including osteoarthritis, rheumatoid arthritis, and periodontitis [3, 12, 13]. Studies have shown that inflammaging is linked to periodontal epithelial barrier dysfunction [14]. Knockout of TLR9 to suppress inflammaging has been found to mitigate alveolar bone resorption [15]. Recent research suggests that AGEs may induce inflammaging [16]. Thus, we hypothesize that AGEs exacerbate periodontitis by inducing inflammaging.

Mounting evidence underscores the role of glycolysis in cellular senescence [17, 18]. Glycolysis is a critical metabolic pathway for cellular energy production. Upon glucose uptake, the PI3K/Akt/mTOR signaling pathway is activated, which upregulates the expression of glucokinase (GK), initiating glucose catabolism [19]. Several factors, including AGEs [20], can redirect cellular metabolism toward glycolysis. Elevated glycolysis has been shown to contribute to inflammatory dysfunction, SASP secretion, and cellular senescence [21, 22]. However, whether glycolysis is involved in AGE-induced inflammaging remains unclear.

In this study, human periodontal ligament fibroblasts (hPDLFs) were isolated and stimulated with AGEs to investigate AGE-induced inflammaging by assessing cellular senescence and SASP secretion. Additionally, the roles of the PI3K/Akt pathway and glycolysis in AGE-induced inflammaging were examined.

## Materials and methods

### AGE-BSA preparation

Bovine serum albumin (BSA, Solebao, Beijing, China) at 50 mg/mL and D-glyceraldehyde (0.1 mol/L, Aladding, Shanghai, China) were dissolved in sterile phosphate buffer (0.2 mol/L, pH 7.4). The mixture was filtered through a 0.22-µm filter membrane and sterilized and then incubated in the dark at 37°C for 7 days. Following incubation, the mixture was dialyzed using phosphate-buffered saline (PBS, pH 7.4) to remove free glyceraldehyde and unbound low molecular weight reactants. As a control, 50 mg/mL BSA without glyceraldehyde was incubated under the same conditions. Given the fluorescent nature of AGEs, the fluorescence intensity of the AGE-BSA solution was measured using a fluorescence spectrophotometer to differentiate between the AGE-BSA and the control solution (excitation wavelength 370 nm, emission wavelength 440 nm, and slit width 3 nm). The fluorescence intensity of the AGE-BSA solution was found to be 45 times higher than that of the control.

### Cell culture and identification

Twenty healthy premolars extracted for orthodontic treatment were collected and stored in a solution containing 100 U/mL penicillin and 100 mg/mL streptomycin. The periodontal ligament tissue was carefully scraped and cut into small pieces. The samples were then digested in type I collagenase (Invitrogen) for 30 minutes, followed by incubation in dulbecco’s modified eagle medium, DMEM supplemented with 15% fetal bovine serum (FBS), 1% 100 U/mL penicillin, and 100 mg/mL streptomycin at 37°C with 5% CO2. Cells were passaged using 0.25% trypsin-ethylene diamine tetraacetic acid, EDTA (Gibco, USA). The third to sixth generations of cells were used in this study. CD105, CD90, CD31, and CD14 were selected for fibroblast cell identification. Patients enrolled in the study were aged 18–25 years and provided signed informed consent. The study was approved by the Ethics Committee of the School of Stomatology at Guizhou Medical University, Ethical Review Document No. 31 of 2021

### Quantitative real‐time polymerase chain reaction

Trizol (Thermo Fisher, USA) was used to extract total RNA from hPDLFs. The obtained RNA was then reverse transcribed into cDNA following the protocol of the PrimeScript RT Reagent Kit (Takara, Japan). Finally, quantitative real‐time polymerase chain reaction (qRT-PCR) was performed to measure the mRNA levels using the TB Green® Premix Ex Taq™ kit. The specific primers are listed below.

**Table:** 

Gene	Forward sequence (5‘-3‘)	Reverse sequence (5‘-3‘)
IL-1β	ATC AGC ACC TCT CAA GCA G	AGT CCA CAT TCA GCA CAG G
IL-6	CAA TAA CCA CCC CTG ACC	GCG CAG AAT GAG ATG AGT
TNF-α	GGA AAG GAC ACC ATG AGC	CCA CGA TCA GGA AGG AGA
β-actin	CCT GGC ACC CAG CAC AAT	GGG CCG GAC TCG TCA TAC

### Western blotting

Proteins were extracted from hPDLFs using a total protein extraction kit (Solarbio, Beijing, China) according to the manufacturer’s protocol. Protein samples from each group were subjected to 10% sodium dodecyl sulfate-polyacrylamide gel electrophoresis (SDS-PAGE) and subsequently transferred to a 0.45 µm polyvinylidene difluoride, PVDF membrane (Millipore) via electroblotting. The membranes were blocked with 5% milk for 1 hour, followed by overnight incubation at 4°C in the dark with primary antibodies against β-actin (1:3,000; Wuhan Sanying), HKII (1:2,000, Abcam, USA), PKM2 (1:2,000, Abcam, USA), GLUT1 (1:1,000, Abcam, USA), LDHA (1:2,000, Abcam, USA), P16 (1:2,000; Wuhan Sanying), P21 (1:2,000; Wuhan Sanying), P53 (1:2,000; Wuhan Sanying), RAGE (1:2,000; Wuhan Sanying), AKT2 (1:2,000; Wuhan Sanying), p-AKT (1:2,000; Wuhan Sanying), mTOR (1:2,000; Wuhan Sanying), and p-mTOR (1:2,000; Wuhan Sanying). The membranes were then incubated with a secondary antibody (1:5,000, Wuhan Sanying) conjugated to horseradish peroxidase (HRP) at room temperature for 1 hour. The bound antibody was detected using the West Pico Chemiluminescent Substrate System (SuperSignal, BioSpectrum®310 Imaging System, USA). The resulting images were analyzed with ImageJ software (National Institutes of Health).

### Measurement of glucose uptake and lactic acid production

The measurement of glucose and lactic acid content in the supernatant was performed according to the protocols of the glucose detection kit (Nanjing Jiancheng, China) and the lactate detection kit (Nanjing Jiancheng, China). The glucose uptake was calculated as follows: glucose content (t0) – glucose content (t1). The lactic acid production was calculated as follows: lactic acid content (t1) – lactic acid content (t0).

### SA-β-Gal staining

SA-β-Gal staining was performed using the SA-β-Gal staining kit (Beyotime, China). Cells were washed three times with PBS and then fixed at room temperature with 4% paraformaldehyde (PFA) for 15 minutes. Following fixation, cells were incubated overnight in the dark at 37°C in a working solution containing 0.05 mg/mL X-gal. After rinsing with PBS, the cells were observed under a microscope at 100x magnification. Five random fields from each sample were selected and analyzed using ImageJ software. SA-β-Gal positive cells were stained blue.

### ELISA

The secretion levels of IL-1β, IL-6, and TNF-α were determined using the respective ELISA kits (Elabscience, China). A volume of 100 μL/well of cell supernatant was added to each well of a 96-well plate and incubated at 37°C for 90 minutes. Following incubation, 100 μL/well of biotinylated detection antibody was added to each well and incubated for 60 minutes. The plate was washed five times, and 100 μL/well of HRP conjugate was added to each well and incubated for 30 minutes. After five additional washes, substrate reagent was added and incubated for 15 minutes, followed by the addition of stop solution. Finally, the optical density (OD) of each well was measured at a wavelength of 450 nm using an enzyme marker.

### Statistical analysis

All tests were conducted in triplicate, and the data are presented as the mean ± standard error of the mean. One-way ANOVA was used to compare differences between groups, with *p* < 0.05 considered statistically significant. All statistical analyses were performed using the Statistical Package for the Social Sciences (SPSS) software (version 20 for Windows, SPSS Inc., USA).

## Results

### AGE-induced inflammaging in hPDLFs

**Figure 1:**
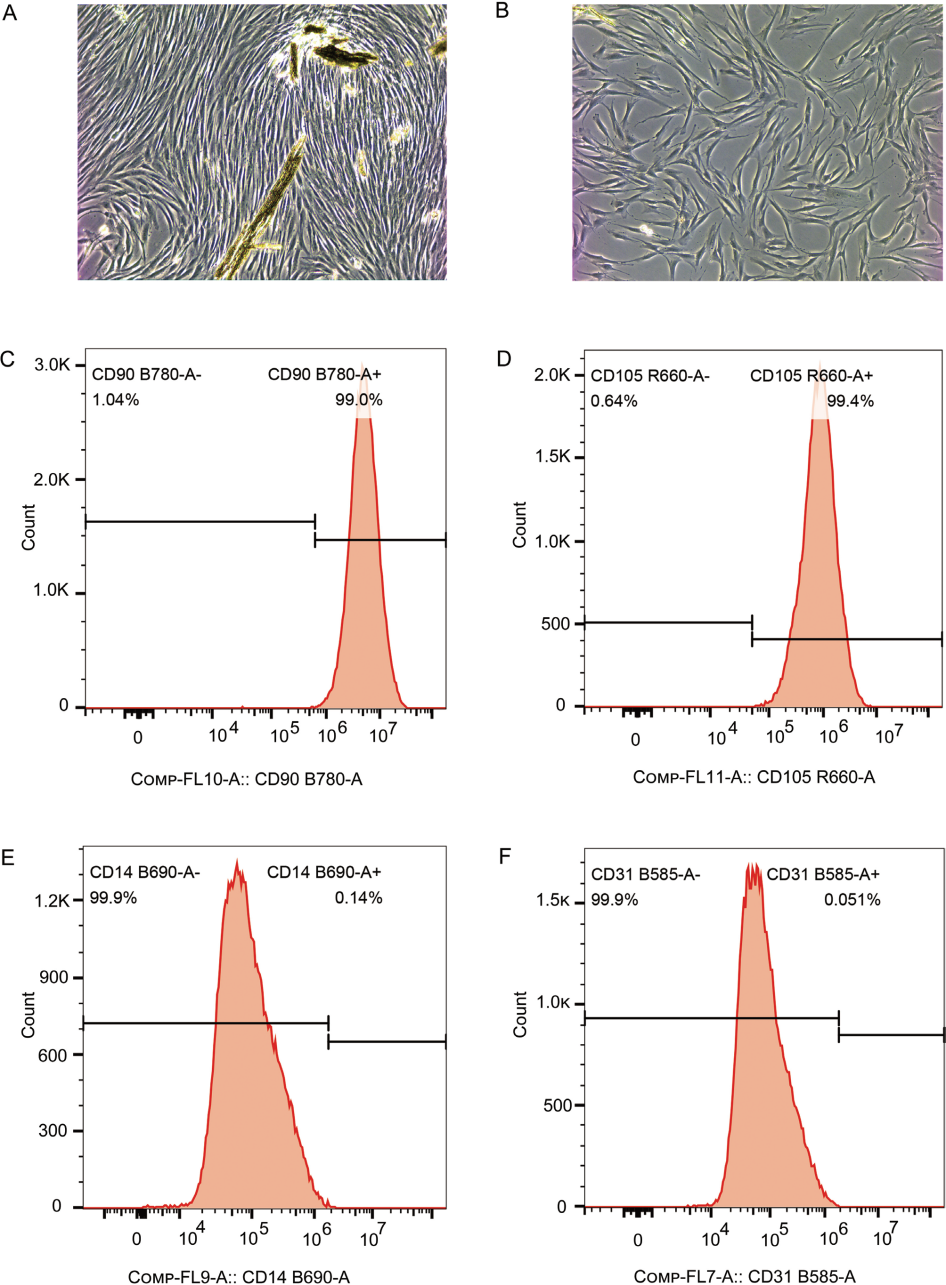
Extraction and identification of hPDLFs. (A) The photo of hPDLFs in generation 1 (100x). (B) The photo of hPDLFs in generation 1 (100x). (C–F) Results of the expression of surface antigens CD90, CD105, CD14, and CD31 analyzed by flow cytometry, respectively. hPDLFs: human periodontal ligament fibroblasts.

hPDLFs were isolated and identified using flow cytometry with antibodies against CD14, CD31, CD90, and CD105, and the results are shown in Figure 1. Figure 1A and 1B displays the long and stringy morphology of hPDLFs at passages p0 and p1. Figure 1C, 1D, 1E, and 1F shows that the percentages of CD90, CD105, CD14, and CD31 positive cells are 99.0, 99.4, 0.14, and 0.051%, respectively. These findings confirm that the cells extracted from periodontal tissue are derived from mesenchymal stem cells, thus identifying them as periodontal ligament fibroblasts.

**Figure 2:**
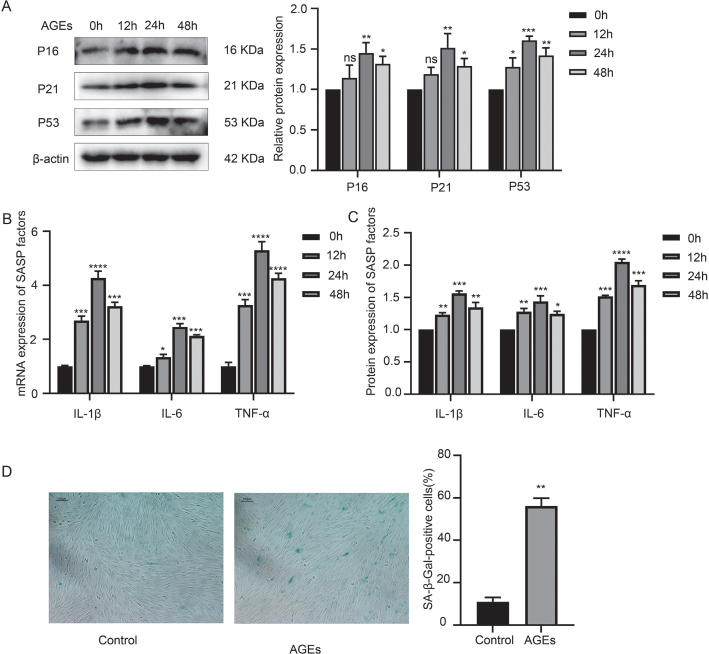
AGE-induced inflammaging in hPDLFs. (A) Western blotting of senescence characteristic proteins of p16, p21, and p53 in the control (oh) group and AGE group (12 h, 24 h, and 48 h). (B) mRNA expression of SASP factors, including IL-1β, IL-6, TNF-α in the control (oh) group and AGE group (12 h, 24 h, and 48 h). (C) Protein expression of SASP factors, including IL-1β, IL-6, and TNF-α in the control (oh) group and AGE group (12 h, 24 h, and 48 h). (D) SA-β-Gal staining in the control and AGE group. AGE: advanced glycation end product; SASP: senescence-associated secretory phenotype; hPDLFs: human periodontal ligament fibroblasts. *p < 0.05, **p < 0.01, ***p < 0.001, and ****p < 0.0001 versus control group.

hPDLFs from p3 to p6 were stimulated with 200 µg/mL AGEs, and the expression of IL-1β, IL-6, TNF-α, p16, p21, p53, and β-galactosidase activity was measured by q-PCR, ELISA, Western blotting (WB), and SA-β-Gal staining, with the results presented in Figure 2. As shown in Figure 2, after AGE administration, the expression levels of SASP factors, including IL-1β, IL-6, TNF-α, as well as p16, p21, and p53, increased gradually, peaked at 24 hours, and then decreased in a time-dependent manner. Additionally, β-galactosidase activity was elevated, suggesting that AGEs could induce inflammaging in hPDLFs.

### Suppression of glycolysis-alleviated AGE-induced inflammaging in hPDLFs

In addition to the inflammaging-related changes in hPDLFs following AGE stimulation, the expression of glycolytic enzymes, including hexokinase II (HKII), pyruvate kinase M2 (PKM2), glucose transporter 1 (GLUT1), and lactate dehydrogenase A (LDHA), significantly increased after 24 hours of 200 µg/mL AGE stimulation. Furthermore, lactate production and glucose uptake were also significantly elevated. These findings suggest that AGEs can enhance glycolysis in hPDLFs.

**Figure 3:**
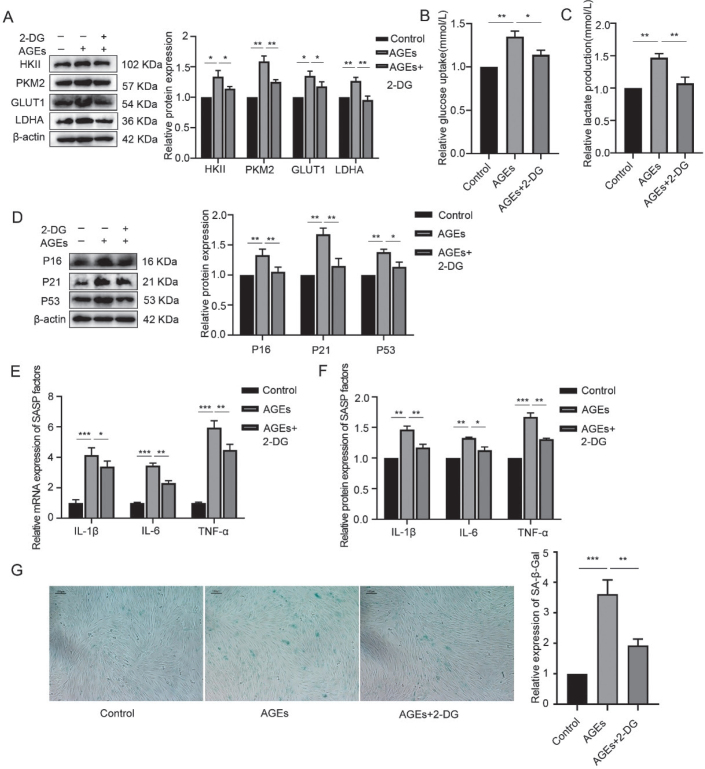
Suppression of glycolysis-alleviated AGE-induced inflammaging in hPDLFs. (A) Western blotting of key enzymes of glycolysis, including HKII, PKM2, GLUT1, LDHA in the control, AGEs, and AGEs+2-DG group. (B) Glucose uptake in the control, AGEs, and AGEs+2-DG group. (C) Lactate production in the control, AGEs, and AGEs+2-DG group. (D) Western blotting of senescence characteristic protein of p16, p21, and p53 in the control, AGEs, and AGEs+2-DG group. (E) mRNA expression of SASP factors including IL-1β, IL-6, and TNF-α in the control, AGEs, and AGEs+2-DG group. (F) Protein expression of SASP factors including IL-1β, IL-6, and TNF-α in the control, AGEs, and AGEs+2-DG group. (G) SA-β-Gal staining in the control, AGEs, and AGEs+2-DG group. AGEs: advanced glycation end products; SASP: senescence-associated secretory phenotype; hPDLFs: human periodontal ligament fibroblasts; 2-DG: 2-deoxyglucose. *p < 0.05, **p < 0.01, and ***p < 0.001.

To further investigate the role of glycolysis in AGE-induced inflammaging, hPDLFs were pretreated with 2-deoxyglucose (2-DG), a glycolytic inhibitor, for 2 hours before AGE stimulation for 24 hours. The results, shown in Figure 3, indicated that 2-DG treatment reduced the expression of HKII, PKM2, GLUT1, and LDHA, as well as lactate production and glucose uptake, demonstrating that 2-DG effectively inhibits glycolysis. Moreover, the expression levels of p16, p21, p53, and β-galactosidase activity were significantly decreased in hPDLFs pretreated with 2-DG and stimulated with AGEs, compared to those exposed to AGE alone. Additionally, the levels of SASP factors, including gene and protein expression, were significantly reduced after 2-DG pretreatment. These results suggest that inhibition of glycolysis can alleviate AGE-induced inflammaging in hPDLFs.

### Inhibition of AKT/mTOR pathway activation downregulated AGE-induced glycolysis and inflammaging

**Figure 4:**
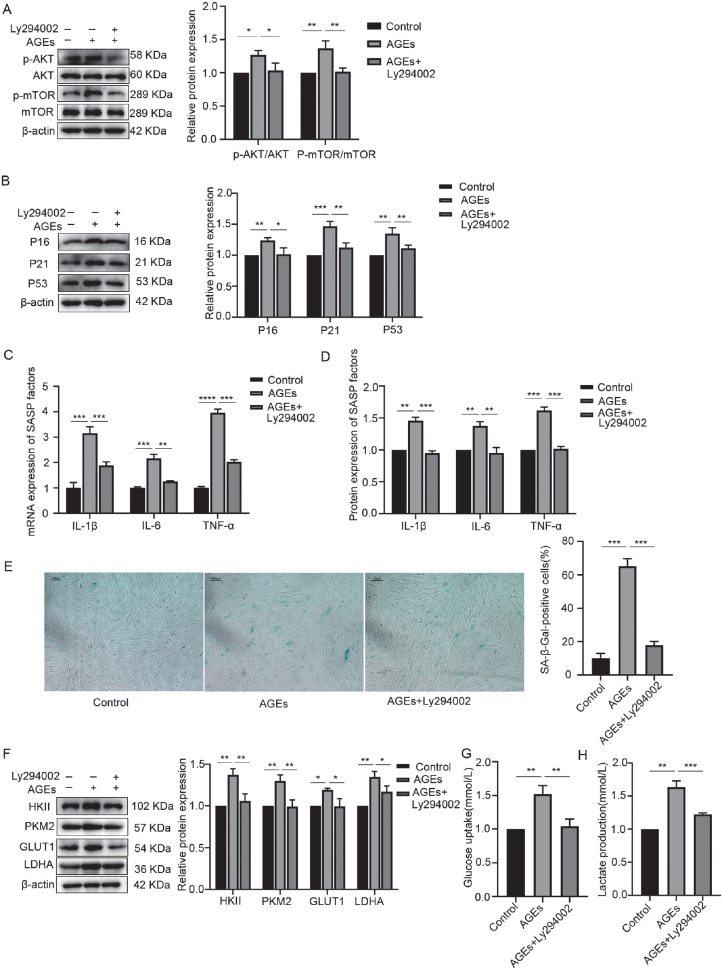
Inhibition of AKT/mTOR pathway activation downregulated AGE-induced glycolysis and inflammaging. (A) Western blotting of AKT, p-AKT, mTOR, p-mTOR in the control, AGEs, and AGEs+Ly294002 group. (B) Western blotting of senescence characteristic protein of p16, p21, and p53 in the control, AGEs, and AGEs+Ly294002 group. (C) mRNA expression of SASP factors including IL-1β, IL-6, and TNF-α in the control, AGEs, and AGEs+Ly294002 group. (D) Protein expression of SASP factors including IL-1β, IL-6, and TNF-α in the control, AGEs, and AGEs+Ly294002 group. (E) SA-β-Gal staining in the control, AGEs, and AGEs+Ly294002 group. (F) Relative OD value of SA-β-Gal staining in each group. (G) Western blotting of key enzymes of glycolysis including HKII, PKM2, GLUT1, and LDHA in the control, AGEs, and AGEs+Ly294002 group. (H) Glucose uptake in the control, AGEs, and AGEs+Ly294002 group. (I) Lactate production in the control, AGEs, and AGEs+Ly294002 group. AGEs: advanced glycation end products; SASP: senescence-associated secretory phenotype; OD: optical density. *p < 0.05, **p < 0.01, and ***p < 0.001.

hPDLFs were treated with 200 µg/mL AGEs for 24 hours, and the activation of the AKT/mTOR signaling pathway was assessed, with the results shown in Figure 4. As illustrated in Figure 4, after AGE administration, the ratio of p-AKT/AKT and p-mTOR/mTOR significantly increased, indicating that AGEs could phosphorylate AKT and mTOR, ultimately activating the AKT/mTOR signaling pathway.

To further explore the role of AKT/mTOR signaling pathway activation in AGE-induced glycolysis and inflammaging, hPDLFs were pretreated with 10 mmol/L Ly294002, an inhibitor of the AKT/mTOR signaling pathway, for 2 hours, followed by stimulation with 200 µg/mL AGEs for 24 hours. The activation of the AKT/mTOR signaling pathway, glycolysis, and inflammaging-related changes were measured, and the results are presented in Figure 4. The ratios of p-AKT/AKT and p-mTOR/mTOR were significantly reduced in hPDLFs pretreated with Ly294002 and stimulated with AGEs compared to those exposed to AGEs alone, confirming that Ly294002 inhibits the activation of the AKT/mTOR signaling pathway. Moreover, the levels of HKII, PKM2, GLUT1, LDHA, as well as lactate production and glucose uptake, were significantly decreased after Ly294002 pretreatment and AGE stimulation, indicating that inhibition of the AKT/mTOR signaling pathway downregulated AGE-induced glycolysis. Additionally, Ly294002 pretreatment significantly reduced the expression levels of SASP factors, as well as p16, p21, p53, and β-galactosidase activity, suggesting that inhibiting the activation of the AKT/mTOR signaling pathway alleviated AGE-induced inflammaging.

### Block of RAGE-inhibited AGE-induced activation of AKT/mTOR pathway, glycolysis, and inflammaging

**Figure 5:**
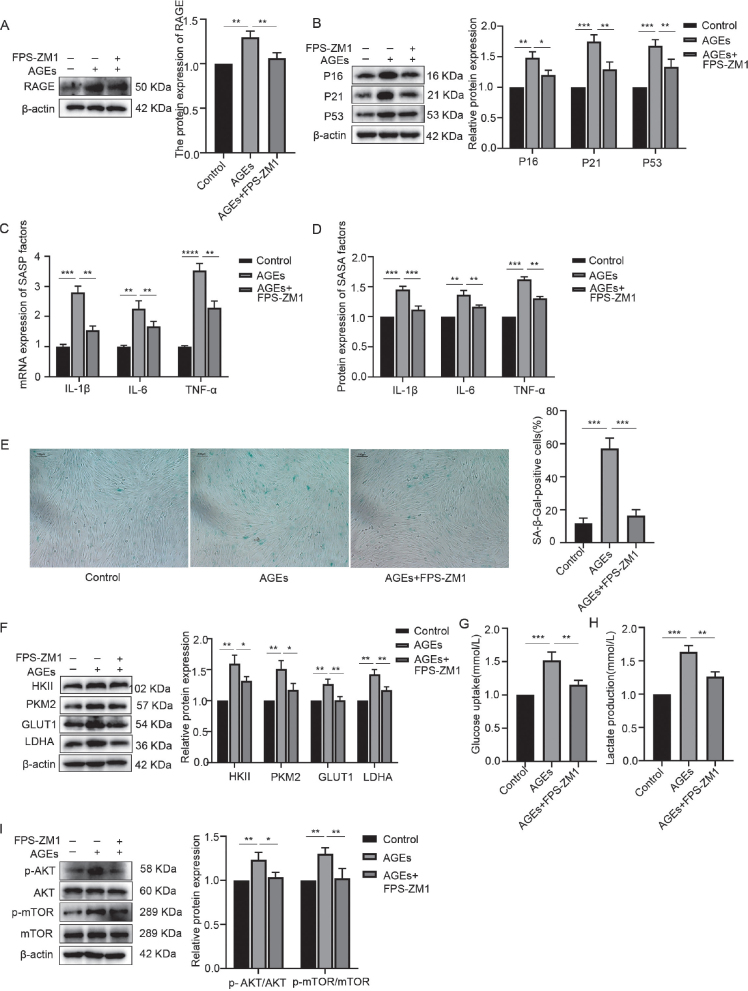
Block of RAGE-inhibited AGE-induced activation of AKT/mTOR pathway, glycolysis, and inflammaging. (A) RAGE expression in the control, AGEs, and AGEs+FPS-ZM1 group. (B) Western blotting of senescence characteristic protein of p16, p21, p53 in the control, AGEs, and AGEs+FPS-ZM1 group. (C) mRNA expression of SASP factors including IL-1β, IL-6, and TNF-α in the control, AGEs, and AGEs+FPS-ZM1 group. (D) Protein expression of SASP factors including IL-1β, IL-6, TNF-α in the control, AGEs, and AGEs+FPS-ZM1 group. (E) SA-β-Gal staining in the control, AGEs, and AGEs+FPS-ZM1 group. (F) Relative OD value of SA-β-Gal staining in each group. (G) Western blotting of key enzymes of glycolysis including HKII, PKM2, GLUT1, LDHA in the control, AGEs, and AGEs+FPS-ZM1 group. (H) Glucose uptake in the control, AGEs, and AGEs+FPS-ZM1 group. (I) Lactate production in the control, AGEs, and AGEs+FPS-ZM1 group. (J) Western blotting of AKT, p-AKT, mTOR, p-mTOR in the control, AGEs, and AGEs+FPS-ZM1 group. RAGE: receptor for advanced glycation end products; AGEs: advanced glycation end products; SASP: senescence-associated secretory phenotype; OD: optical density. *p < 0.05, **p < 0.01, and ***p < 0.001.

hPDLFs were treated with 200 µg/mL AGEs for 24 hours, and the expression level of the RAGE was assessed via WB. The results, shown in Figure 5, indicate that exposure to 200 µg/mL AGEs resulted in a noticeable increase in RAGE expression. Moreover, when 500 nmol/L FPS-ZM1, an antagonist of RAGE, was added to block RAGE, the AGE-induced increase in RAGE expression was significantly suppressed, suggesting that AGEs induce RAGE expression in a positive feedback manner.

To further investigate the role of RAGE in AGE-induced changes in hPDLFs, 500 nmol/L FPS-ZM1 was used to block RAGE, followed by stimulation with 200 µg/mL AGEs for 24 hours. The results, shown in Figure 5, revealed that compared to the AGE stimulation group, the levels of inflammaging-related markers, glycolysis-related markers, and phosphorylation of AKT and mTOR were significantly reduced in the AGEs plus FPS-ZM1 group. This indicates that blocking RAGE inhibited the AGE-induced activation of the AKT/mTOR pathway, glycolysis, and inflammaging.

### AGEs significantly promote lipopolysaccharide-induced secretion of inflammatory factors

**Figure 6:**
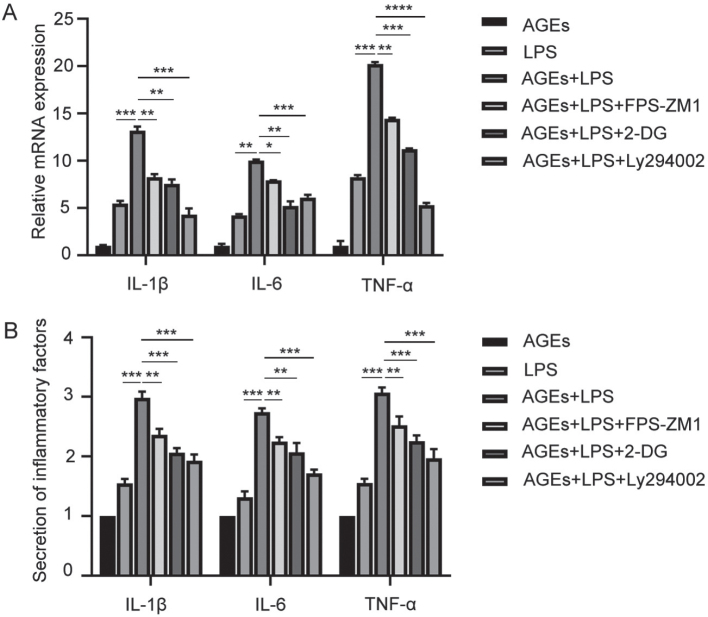
AGEs significantly promote LPS-induced secretion of inflammatory factors. (A) mRNA expression of inflammatory factors including IL-1β, IL-6, and TNF-α in the AGEs, LPS, AGEs+LPS, AGEs+LPS+FPS-ZM1, AGEs+LPS+2-DG, and AGEs+LPS+Ly294002 group. (B) Secretion of inflammatory factors including IL-1β, IL-6, TNF-α in the AGEs, LPS, AGEs+LPS, AGEs+LPS+FPS-ZM1, AGEs+LPS+2-DG, and AGEs+LPS+Ly294002 group. AGEs: advanced glycation end products; LPS: lipopolysaccharide; 2-DG: 2-deoxyglucose. *p < 0.05, **p < 0.01, ***p < 0.001, and ****p < 0.0001.

hPDLFs were pretreated with 200 µg/mL AGEs for 12 hours and then stimulated with lipopolysaccharide (LPS) for 24 hours, after which the secretion of pro-inflammatory cytokines was measured. The results, shown in Figure 6, indicate that pro-inflammatory cytokine secretion was significantly higher in the group co-treated with AGEs and LPS compared to the group with LPS stimulation alone, suggesting that AGEs promote LPS-induced inflammation.

To further explore the possible mechanism underlying the promotion of LPS-induced inflammation by AGEs, FPS-ZM1, LY294002, and 2-DG were co-administered with AGEs plus LPS. All three inhibitors downregulated the pro-inflammatory cytokine secretion induced by AGEs and LPS (Figure 6), indicating that RAGE, the AKT/mTOR pathway, and glycolysis are involved in the AGE-mediated promotion of LPS-induced inflammation.

## Discussion

In this study, we found that AGEs could induce inflammaging, which may be linked to the increased inflammatory response in hPDLFs stimulated by LPS. We also uncovered the roles of glycolysis and the RAGE/AKT/mTOR signaling pathway in AGE-induced inflammaging. These findings highlight the involvement of inflammaging in the AGE-enhanced inflammation triggered by LPS, potentially offering new strategies for the treatment of periodontitis aggravated by AGEs.

Periodontitis is an age-related disease, and inflammaging has been shown to be closely associated with its progression [3]. Previous studies have demonstrated that inflammaging-induced epithelial barrier dysfunction plays a key role in the exacerbation of periodontitis, particularly in the context of diabetes [14]. In terms of inflammaging, the characteristic proteins of senescence, SASP factors, and positive staining for SA-β-gal are considered the best markers for identifying inflammaging in cells [23]. The periodontal ligament is a critical site for the host’s immune response to external pathogen stimulation [24], with periodontal ligament fibroblasts (PDLFs) playing an essential role in this process. Overexpression of pro-inflammatory cytokines in PDLFs can amplify local inflammatory responses and mediate periodontal tissue damage by continuously triggering immune responses [25].

In this study, hPDLFs were stimulated with AGEs, and we found that AGEs promoted the expression of characteristic senescence proteins, including p16, p21, and p53, SASP factors such as IL-1β, IL-6, and TNF-α, and increased the number of cells with positive SA-β-gal staining. Antagonizing AGEs binding to RAGE downregulated these inflammaging markers, indicating that AGEs can induce inflammaging in hPDLFs. As periodontitis is triggered by bacterial infection, with LPS being a major virulence factor in periodontitis, LPS stimulation is commonly used as an in vitro model to study periodontitis [26]. In this study, hPDLFs were pretreated with AGEs and then stimulated with LPS to simulate the role of AGEs in periodontitis. The results showed that AGEs enhanced the expression of pro-inflammatory cytokines induced by LPS. Collectively, these findings suggest that AGEs can induce inflammaging and exacerbate periodontitis, with inflammaging potentially serving as the mechanism by which AGEs aggravate the disease.

The molecular mechanisms underlying AGE-induced inflammaging in hPDLFs remain unclear. Recent studies have shown that senescence is often accompanied by alterations in energy metabolism [17, 27], with glycolysis, a key energy metabolism pathway, being closely linked to SASP secretion and cellular senescence [22, 28]. Evidence suggests that PKM2, a rate-limiting enzyme in glycolysis, plays a role in SASP and senescence [21, 29], and that inhibiting glycolysis can reduce β-galactosidase activity and suppress senescence [30]. On the other hand, in γ-ray-induced senescent cells, the glycolytic flux is significantly elevated [31]. The pro-inflammatory cytokine IL-6, a component of the SASP, directs glucose metabolism toward glycolysis [32]. Additionally, TNF-α, another important SASP component, induces metabolic reprogramming that favors glycolytic activity [33]. Collectively, these findings suggest that glycolysis promotes cellular senescence, and senescence, in turn, reinforces glycolytic metabolism. In this study, we found that AGEs increased glucose uptake and lactate production, along with the expression levels of PKM2 and other key glycolytic enzymes, including HKII, GLUT1, and LDHA. These results were consistent with previous studies [20] and suggest that AGEs shift cellular metabolism toward glycolysis. To further investigate the role of glycolysis in AGE-induced inflammaging, we co-administered 2-DG, a glycolysis inhibitor, with AGEs. The results showed that inhibiting glycolysis attenuated the expression levels of inflammaging-related factors induced by AGEs. These findings indicate that AGEs induce inflammaging by promoting glycolysis. Further studies are warranted to comprehensively evaluate the role of inflammaging in AGE-induced glycolysis promotion, and the interplay between glycolysis and inflammaging may influence the identification of potential therapeutic targets for AGE-related diseases.

However, the mechanism by which AGEs promote glycolysis remains unclear. Many studies have demonstrated that the AKT/mTOR signaling pathway plays a crucial role in regulating glycolysis [34, 35], with inhibition and over-activation of the AKT/mTOR pathway suppressing and promoting glycolysis, respectively [36, 37]. The results of this study showed that AGEs could phosphorylate AKT and mTOR to activate the AKT/mTOR signaling pathway. Inhibition of AKT/mTOR signaling by Ly294002 reduced glycolysis and inflammaging induced by AGEs, confirming that the AKT/mTOR pathway is involved in AGE-induced glycolysis and inflammaging. Finally, a RAGE antagonist was used to explore the role of RAGE in AGE-induced inflammaging, glycolysis, and activation of the AKT/mTOR signaling pathway. As is well known, AGEs primarily regulate cell metabolism by binding to RAGE [7]. The results of this study showed that the RAGE antagonist could downregulate the activation of the AKT/mTOR pathway, glycolysis, and inflammaging, suggesting that AGEs bind to RAGE to activate the AKT/mTOR pathway, increase glycolysis, and induce inflammaging.

In summary, it can be concluded that AGEs bind to RAGE to activate the AKT/mTOR signaling pathway, which shifts cell metabolism toward glycolysis and ultimately induces in-flammaging. At the same time, AGEs enhance the inflammation induced by LPS. We propose a potential link between inflammaging and the increased inflammation in periodontitis aggravated by AGEs. To date, there are few therapeutic strate-gies available for AGE-aggravated periodontitis. Therefore, the findings of this study provide an experimental basis for the prevention and treatment of AGE-aggravated periodontitis by targeting inflammaging through the RAGE/AKT/mTOR pathway and glycolysis. However, further research is needed to fully elucidate the specific mechanisms by which AGEs contribute to periodontitis.

## Conclusion

Our study indicated that AGEs induced inflammaging through binding to RAGE to activate AKT/mTOR pathway and eventually enhancing glycolysis level, which may contribute to the increased inflammatory response triggered by LPS. These findings suggest that inflammaging is a critical mechanism through which AGEs exacerbate periodontitis.
